# Open access: changing the way chemistry is published

**DOI:** 10.1186/1758-2946-4-S1-P50

**Published:** 2012-05-01

**Authors:** Jan Kuras, Bailey Fallon

**Affiliations:** 1 Chemistry Central, London WC1X 8HL, UK

## 

Publishing research in open access (OA) journals ensures free and permanent unrestricted online access to peer-reviewed articles [1], and increased visibility for articles and increased potential citations. In addition, authors retain copyright to their work, and data can be redistributed, reused and translated freely [2]. Removing barriers to accessing and sharing data is beneficial for researchers and their institutions, as well as funders, educators and the public.

Having had success in biomedical and physics publishing, support continues to grow for open access in chemistry, in particular amongst the cheminformatics community. The range of benefits for authors and institutions makes open access a viable and effective publishing model for chemistry research.

This poster provides an overview of the benefits of open access, and compares the OA model to traditional subscription journal publishing.
